# Proteomic characterization of high-density lipoprotein particles in patients with non-alcoholic fatty liver disease

**DOI:** 10.1186/s12014-018-9186-0

**Published:** 2018-03-06

**Authors:** Prahlad K. Rao, Kate Merath, Eugene Drigalenko, Avinash Y. L. Jadhav, Richard A. Komorowski, Matthew I. Goldblatt, Anand Rohatgi, Mark A. Sarzynski, Samer Gawrieh, Michael Olivier

**Affiliations:** 1 Department of Genetics, Texas Biomedical Research Institute, San Antonio, TX USA; 2 Biotechnology and Bioengineering Center, Medical College of Wisconsin, Milwaukee, WI USA; 3 Present Address: Department of Pediatrics, University of Tennessee Health Science Center, Memphis, TN 38103 USA; 4 Present Address: Center for Precision Medicine, Wake Forest School of Medicine, Winston-Salem, NC USA; 5 Department of Pathology, Froedtert and Medical College of Wisconsin, Milwaukee, WI USA; 6 Department of Surgery, Froedtert and Medical College of Wisconsin, Milwaukee, WI USA; 7 Department of Internal Medicine, University of Texas Southwestern Medical Center, Dallas, TX USA; 8 Department of Exercise Science, University of South Carolina, Columbia, SC USA; 9 Department of Medicine, Indiana University, Indianapolis, IN USA

**Keywords:** High-density lipoproteins, Proteomics, Non-alcoholic fatty liver disease, Obesity, Anti-thrombotic

## Abstract

**Background:**

Metabolic diseases such as obesity and diabetes are associated with changes in high-density lipoprotein (HDL) particles, including changes in particle size and protein composition, often resulting in abnormal function. Recent studies suggested that patients with non-alcoholic fatty liver disease (NAFLD), including individuals with non-alcoholic steatohepatitis (NASH), have smaller HDL particles when compared to individuals without liver pathologies. However, no studies have investigated potential changes in HDL particle protein composition in patients with NAFLD, in addition to changes related to obesity, to explore putative functional changes of HDL which may increase the risk of cardiovascular complications.

**Methods:**

From a cohort of morbidly obese females who were diagnosed with simple steatosis (SS), NASH, or normal liver histology, we selected five matched individuals from each condition for a preliminary pilot HDL proteome analysis. HDL particles were enriched using size-exclusion chromatography, and the proteome of the resulting fraction was analyzed by liquid chromatography tandem mass spectrometry. Differences in the proteomes between the three conditions (normal, SS, NASH) were assessed using label-free quantitative analysis. Gene ontology term analysis was performed to assess the potential impact of proteomic changes on specific functions of HDL particles.

**Results:**

Of the 95 proteins identified, 12 proteins showed nominally significant differences between the three conditions. Gene ontology term analysis revealed that severity of the liver pathology may significantly impact the anti-thrombotic functions of HDL particles, as suggested by changes in the abundance of HDL-associated proteins such as antithrombin III and plasminogen.

**Conclusions:**

The pilot data from this study suggest that changes in the HDL proteome may impact the functionality of HDL particles in NAFLD and NASH patients. These proteome changes may alter cardio-protective properties of HDL, potentially contributing to the increased cardiovascular disease risk in affected individuals. Further validation of these protein changes by orthogonal approaches is key to confirming the role of alterations in the HDL proteome in NAFLD and NASH. This will help elucidate the mechanistic effects of the altered HDL proteome on cardioprotective properties of HDL particles.

**Electronic supplementary material:**

The online version of this article (10.1186/s12014-018-9186-0) contains supplementary material, which is available to authorized users.

## Background

Metabolic disorders in humans, such as obesity or diabetes, are often associated with liver abnormalities, including non-alcoholic fatty liver disease (NAFLD). NAFLD defines a spectrum of pathologies from hepatic steatosis to nonalcoholic steatohepatitis (NASH), characterized by the additional occurrence of lobular inflammation, hepatocellular ballooning and perisinusoidal or pericullular fibrosis [1]. NASH can lead to liver cancer or cirrhosis, and may require liver transplantation. Patients with NAFLD and NASH show increased risk for cardiovascular disease (CVD), potentially mediated by obesity, elevated plasma triglyceride and low density lipoprotein (LDL) cholesterol levels, and altered high-density lipoprotein (HDL) cholesterol levels, reflecting an overall atherogenic lipid profile [2].

HDL particles serve multiple essential functions. Apart from reverse cholesterol transport (RCT) promoting lipid efflux from cells, they also contain proteins which function as acute-phase response proteins and impart tissue-protective anti-inflammatory, anti-oxidative and anti-thrombotic properties which are also anti-atherogenic [3]. In obese and diabetic individuals, plasma levels of HDL-C are often reduced, with a preponderance of small, dense HDL particles. This is further exacerbated in patients with NAFLD and NASH [4]. It has been proposed that these particles are dysfunctional increasing the risk for atherosclerosis [5].

Proteomics has been used previously to examine HDL particle composition in patients with cardiovascular disease pathologies. Analysis of HDL particles from patients with coronary artery disease (CAD) patients revealed an enrichment of APOE, APOC-IV, PON-1, complement C3 and APOA-IV in HDL particles when compared to healthy controls [6]. Recent studies described increases in abundances of serum amyloid A, C3 and inflammatory proteins in CAD which suggested a shift from an anti-inflammatory role to a pro-inflammatory state of HDL particles [7, 8]. These studies suggest that specific protein alterations in HDL particles may lead to altered HDL function.

To date, no studies have investigated changes in the HDL proteome in NASH or NAFLD, even though HDL particle size has been reported to change in NAFLD patients [4]. Several studies have examined proteomic differences in serum or liver samples between controls and patients with different degrees of NAFLD to identify possible biomarkers for progression of NAFLD [9]. However, specific proteomic changes in HDL particles have not been examined. In this study, we explored whether differences in HDL-associated proteins could potentially point to alterations in HDL functions in patients with NAFLD and NASH. From our clinical cohort, we selected five individuals with normal liver histology, five individuals with steatosis, and five individuals with NASH. Individuals were matched by age and BMI. The HDL proteome was characterized using high-resolution mass spectrometry (MS) after enrichment of HDL particles from serum. To minimize the amount of serum required for analysis, we used size exclusion chromatography (SEC) to enrich lipoprotein particles [10]. Comparing the HDL proteome between normal, SS and NASH subjects, we detected nominally significant quantitative differences in HDL proteins. Gene ontology analysis revealed that proteins potentially affecting anti-thrombotic functions were decreased with increased disease severity. This change in putative HDL-associated proteins may contribute to increased tissue injury and cardiovascular disease risk in NAFLD patients, thereby negatively impacting patients diagnosed with NAFLD.

## Methods

### Recruitment and sample collection

The study protocol was approved by the Medical College of Wisconsin’s Institutional Review Board. Subjects gave written informed consent for participation in the study. Subjects were females of Northern European descent, morbidly obese (BMI ≥ 40 kg/m^2^ or > 35 kg/m^2^ with significant co-morbidities) with documented unsuccessful dietary attempts to lose weight; and who underwent bariatric surgery. A liver biopsy was collected intra-operatively from all patients for histological phenotyping. Patients with alcohol intake > 20 gm/day and those with other liver diseases (hepatitis B, hepatitis C, auto-immune hepatitis, primary biliary cirrhosis, Wilson’s disease, alpha-1 antitrypsin deficiency, or hemochromatosis) based on positive serological tests and suggestive liver histology were excluded. Patients using drugs associated with NAFLD (systemic glucocorticosteroids, Tamoxifen, Tetracycline, Amiodarone, Methotrexate, Valproic Acid, anabolic steroids, estrogens at doses higher than those used for hormone replacement, or other known hepatotoxins) were also excluded. Fasting blood samples for serum extraction, and clinical and biochemical data were collected from all subjects in the morning of the scheduled surgery.

### Histological evaluation and diagnosis

All liver biopsy samples were read by an expert pathologist (R.K.) to define the NAFLD phenotype and semi-quantitatively score the individual histological features and subphenotypes including steatosis, lobular and portal inflammation, hepatocellular ballooning, Mallory’s hyaline and fibrosis according to the scoring system of the NIH NASH Clinical Research Network working group [11]. Subjects with 0–5% macrosteatosis were diagnosed as non-NAFLD controls. NAFLD was diagnosed when ≥ 5% macrosteatosis was present.

Using a strict pathologic protocol based on Dixon [12] to define NASH, each liver biopsy specimen was classified as: (1) simple steatosis alone, (2) possible NASH (> 5% steatosis plus one of the following zone 3 centrilobar findings: lobular inflammation, hepatocyte ballooning with or without Mallory’s hyaline, pericellular/perisinusoidal fibrosis), (3) definite NASH (> 5% steatosis plus two of the following zone 3 centrilobar findings: lobular inflammation, hepatocyte ballooning with or without Mallory’s hyaline, pericellular/perisinusoidal fibrosis), or (4) normal. Patients classified into groups 2 and 3 were combined for the purposes of this analysis. NAFLD was defined as the combination of classifications (1)–(3), covering the complete spectrum from SS to NASH.

### Separation of serum lipoprotein particles and Nano-HPLC–MS/MS

HDL particles were enriched by SEC, as previously described [10] from 100 μl aliquots of serum for each injection. Fractions positive for Apo-AI and in the expected size range for HDL particles were combined and processed for delipidation by extraction with methanol-chloroform to isolate HDL particle-associated proteins. Proteins were quantified and prepared for MS analysis as described before [13].

Protein digests were analyzed on a ThermoFinnigan LTQ ion trap mass spectrometer (Orbitrap Velos) interfaced with a nano-LC system (Waters) equipped with an autosampler through which samples were loaded onto a C_18_ capillary column (15 × 0.75 mm). The capillary column was packed in-house with 5 μm C_18_ RP particles (New Objective, Woburn, MA, USA). Solvents *A* and *B* used for the chromatographic separation were 5% acetonitrile in 0.1% formic acid and 95% acetonitrile in 0.1% formic acid, respectively. Samples were resolved at a rate of 0.3 μl/min using a gradient of 2% B for 0–10 min, 2–40% B from 10 to 50 min, 40–98% B from 50 to 60 min, 2% B from 60 to 65 min and 2% B from 65 to 120 min. Each HDL-containing fraction from an individual serum sample was injected three times as technical replicates to maximize protein identifications.

### Analysis of the serum proteome by nano-HPLC–MS/MS

Serum proteins from individual samples were precipitated, dissolved in Tris buffer, and quantified. 200 μg of proteins were reduced with TCEP, alkylated using iodoacetic acid and digested using LysC-trypsin enzyme mixture (Promega). Peptides were separated on a 50 cm C18 column attached to Dionex Ultimate 3000 nano-UPLC system coupled to Q-Exactive HF hybrid Quadrupole-Orbitrap Mass Spectrometer (Thermo Scientific, Rockford, IL, USA). Good chromatographic separation was observed with a linear gradient consisting of mobile phases A (water with 0.1% formic acid) and B (acetonitrile with 0.1% formic acid) where the gradient was from 5% B at 0 min to 40% B at 80 min. MS spectra were acquired by data dependent scans consisting of MS/MS scans of the twenty most intense ions from the full MS scan with dynamic exclusion option which was 10 s.

### Data analysis

All 45 data files (3 technical MS replicates per sample) were searched against the human Uniprot canonical and isoform database (release 2016_03) using MaxQuant ver. 1.5.3 [14]. Proteins were identified with 1% protein false discovery rate (FDR), determined empirically by reversed decoy database searching according to standard MS analysis approaches. Cysteine carbamidomethylation (+ 57.021) was considered as fixed modification, and oxidation of methionine (+ 15.995) and N-terminal acetylation (+ 42.010) were considered as variable modifications. The main search peptide tolerance was kept at 4.5 ppm and the minimum intensity threshold was kept at 500. The MS/MS match tolerance was kept at 0.5 Da. The remaining settings in MaxQuant were kept at default. The ‘match between runs’ feature was activated.

Label free normalization and quantitation was performed using the LFQ feature of MaxQuant (MaxLFQ) [15]. The minimum number of neighbors was kept at three and average number of neighbors was 6 for LFQ. Data cleaning and statistical analysis was performed using Perseus ver. 1.5.3 [16]. Proteins identified as decoy or contaminants were manually removed. The LFQ values were log-transformed and filtered, with a minimum of 66% of values present for each sample. Missing values were replaced using values computed from the normal distribution with a width of 0.3 and a downshift of 1.8. The minimum number of peptides per identified protein after data clean-up was two peptides per protein.

Analysis of the serum proteome data was carried out in Proteome Discoverer. Spectra were searched using Sequest HT algorithm within the Proteome Discoverer v2.1 (Thermo Scientific) in combination with the human UniProt protein FASTA database (20,193 entries, December 2015). Search parameters were as follows: FT-trap instrument, parent mass error tolerance of 10 ppm, fragment mass error tolerance of 0.02 Da (monoisotopic), variable modifications of 16 Da (oxidation) on methionine and fixed modification of 58 Da (carboxymethylation) on cysteine. Peptide spectral match (PSM) numbers for each identified peptide were scaled using total PSM in a particular sample and normalized via z-score normalization. Welch’s *t* test was used on protein entries with a minimum of three valid values per group to identify proteins that differed significantly (*p* < 0.05) between the normal and NASH samples.

We used statistical regression analysis using R ver. 3.0.3 to examine the association of traits with protein abundance. Function lm() provides the coefficient, and function anova() gives the *p* value of the coefficient.

For gene ontology analysis related to biological process (BP) or molecular function (MF), Consensus Path DB-human [17] was used.

## Results

Our study focused on a group of 15 morbidly obese females for an exploratory HDL proteomics analysis, 5 with no abnormal liver histology, 5 with SS and 5 with NASH. The three groups ranged in age from 45 to 59 years. Individuals were selected by matching the age and BMI between individuals across all three pathological classes (Table 1).

**Table 1:** Summary of clinical characteristics for the 15 individuals described in this study

Characteristic	Control N = 5	SS N = 5	NASH N = 5	*p*
Age (year)	49.8 (5.8)	49.2 (2.4)	51.2 (3.9)	0.72
BMI (kg/m^2^)	50.18 (7.2)	49.88 (6.1)	48.48 (4.7)	0.99
ALT U/L	15.4 (4.21)	25.2 (17.51)	25.4 (18.17)	0.37
Glucose (mg/dl)	98.6 (16.6)	144.2 (72.8)	118.8 (28.3)	0.13
Insulin (mu/ml)	20.06 (6.24)	18.81 (17.55)	28.06 (14.75)	0.73
HOMA	4.91 (1.81)	7.106 (10.01)	8.88 (6.24)	0.72
TChol (mg/dl)	160.8 (23.28)	157 (32.09)	188.25 (49.92)	0.69
TG (mg/dl)	90 (23.18)	173.6 (98.45)	191.75 (82.61)	0.18
HDL (mg/dl)	50.4 (6.06)	39.2 (15.51)	43.25 (6.94)	0.12
LDL (mg/dl)	92.4 (29.19)	93 (25.41)	106.75 (38.74)	0.91
LDL Med (nm)	25.98 (0.66)	26.69 (0.51)	26.19 (0.66)	0.07
HDL Med (nm)	8.54 (0.14)	8.74 (0.15)	8.49 (0.11)	0.02

Values presented are mean (SD). Median LDL sizes were not found to be significantly different in subjects diagnosed with SS or NASH. However, median HDL size was found to be significant between the three groups. *p* value represents difference in mean values between the three conditions

Of the 5 patients diagnosed with NASH, 2 patients had diabetes. All the five patients from the NASH group were also diagnosed with hypertension while only one patient with SS and three patients in the normal liver group were diagnosed with hypertension. None of the patients used sulfonylureas, statins or insulin. One patient in the NASH group indicated the use of metformin while another subject in the NASH group reported the use of thiazolidinediones (TZDs).

No statistically significant differences were noted between the group with normal liver and patients with SS or NASH in age and BMI. Alanine transaminase (ALT), glucose, and triglyceride levels, and homeostatic model assessment (HOMA) showed no significant differences between the three groups. While HDL and LDL levels did not vary significantly between the three groups, the median HDL sizes showed statistically significant difference (*p* < 0.05) between the three groups (Table 1).

### HDL proteome analysis

HDL-enriched fractions isolated by FPLC-SEC were combined, delipidated, proteins digested with trypsin and analyzed by MS. We have previously shown that this protocol is able to perform an in-depth analysis of the HDL-associated proteome from very small serum volumes [10].

MS analysis identified a total of 125 proteins in the 15 samples. On an average, we identified 121 proteins in samples from subjects with normal livers, 120 proteins in subjects with SS, and 122 proteins in subjects diagnosed with NASH.

No proteins were uniquely identified in individuals with any one of the three liver diagnoses, suggesting that any proteomic differences in HDL particles in NAFLD patients are likely to be quantitative.

Therefore, we examined the quantitative differences between proteins that were identified in samples from normal, SS and NASH patients. 95 proteins (FDR < 0.01) were identified across all samples with a minimum of two peptides and a minimum of one unique peptide, and these proteins were used for further analysis (Additional file 1: Supplementary Table). Of these, 60 proteins are annotated as HDL-associated proteins when compared against HDL proteome watch list [18].

12 proteins showed differences in relative abundance between normal, SS and NASH patients (*p* ≤ 0.05). Seven of the 12 proteins were related to endopeptidase function. Alpha-2-macroglobulin (A2MG) and apolipoprotein B (APOB) were increased in SS and NASH and followed the trend NASH > SS > normal. The remaining 10 proteins were decreased in SS and NASH conditions (Table 2).

**Table 2:** Proteins altered significantly between different conditions

Proteins	Normal versus SS versus NASH *p* value	SS versus NASH *p* value	Normal versus NAFLD *p* value
A1BG	0.03	NS	< 0.01
A2MG*	< 0.01	NS	< 0.01
AACT	< 0.01	NS	< 0.01
ANT3	< 0.01	NS	0.018
APOB*	0.01	NS	NS
CBG	< 0.01	NS	< 0.01
CFAB	0.01	NS	< 0.01
CFAI	NS	NS	0.019
FA12	NS	NS	0.01
FETUA	0.03	NS	0.01
HBA1/HBA2	NS	0.04	NS
HEMO	0.04	NS	< 0.01
HEP2	NS	NS	0.04
HRG	0.01	NS	< 0.01
HV323	NS	0.03	NS
IGHG1	NS	NS	0.03
IGHG2	NS	0.05	NS
IGHG3	NS	0.05	0.01
IGHM	NS	0.02	NS
ITIH1	NS	NS	0.03
ITIH2	NS	NS	0.01
KV139	NS	<0.01	NS
KV401	NS	<0.01	NS
LV106	NS	NS	0.04
LV302	NS	NS	0.01
PLMN	0.02	NS	< 0.01
TRFE	0.01	NS	< 0.01

Proteins marked with ‘*’ show increased relative abundance in NASH compared to simple steatosis (SS) and normal conditions

*NS* indicates not significant

When we compared HDL protein abundances between SS and NASH, 7 proteins were nominally significantly changed (Table 2). Six of the seven proteins belong to the immunoglobulin class and were significantly increased in SS.

While all detected protein changes were nominally statistically significant, the observed changes were small. APOB and A2MG abundance in control individuals (morbidly obese females with no liver pathology) was reduced by 5 and 4%, respectively, when compared to HDL particles from morbidly obese NASH subjects. As shown in Table 2, overall, the comparison of protein abundances of HDL particles between normal and NAFLD (SS *plus* NASH) identified 20 proteins that were significantly altered (*p* ≤ 0.05).

### HDL proteins of NASH patients may impact anti-thrombotic functions of HDL

**Fig. 1:**
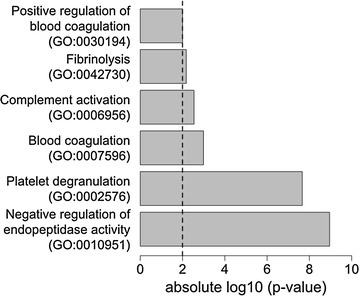
Over-represented GO terms in the biological process (BP) category. Protein abundances are compared between normal and NAFLD (SS *plus* NASH). 18 proteins have been mapped in the BP category. The vertical line indicates *p* value of 0.01

To understand the putative functional implications of altered protein abundances in NAFLD, we performed a GO term over-representation analysis. Analysis of the biological process (BP) GO category revealed the terms ‘negative regulation of endopeptidase activity’ (GO0010951), ‘platelet degranulation’ (GO0002576), ‘blood coagulation’ (GO0007596), “complement activation” (GO0006956), “fibrinolysis” (GO0042730) and “positive regulation of blood coagulation” (GO0030194) to be significantly over-represented (*p* < 0.01, Fig. 1).

Under the Molecular Function (MF) category GO terms, the only over-represented group was “endopeptidase inhibitor activity” (*p* < 0.01).

We further examined abundances of proteins related to the thrombotic function of HDL to analyze the relation between their relative levels between normal versus NAFLD and HDL function. Three proteins implicated in the regulation of blood coagulation (antithrombin III or ANT3, plasminogen or PLMN, histidine-rich glycoprotein or HRG) were significantly decreased in SS and NASH patients compared to individuals with normal liver pathology (*p* ≤ 0.02, Table 2).

### Comparison of HDL proteome to serum protein abundance

We then sought to ascertain that the differences in protein abundances are likely originating from proteins that are HDL-associated and not a reflection of the systemic abundance differences in the serum proteome between the groups that happen to co-elute in the chromatography. The serum proteome of the subjects who were not diagnosed with liver disease was compared with the proteome of subjects diagnosed with NASH. We identified 173 proteins between the two sets of samples. After filtering for proteins which were detected in at least three samples per group, we did not detect any proteins that were significantly different between the two groups.

We also specifically looked at proteins that were identified in the HDL proteome analysis with anti-thrombotic functions (ANT3, PLMN and HRG). We found that these three proteins were not found to be significantly different between the two groups (Table 3).

**Table 3:** Proteins identified in serum of Normal and NASH subjects with functional roles in coagulation

Proteins	Normal	NASH	*p* value
ANT3	9.8 (0.97)	8.4 (1.25)	0.40
PLMN	18.4 (2.14)	23 (2.97)	0.24
HRG	5.2 (0.58)	5.6 (1.21)	0.77

Proteins identified in the analysis of serum proteome that have a role in coagulation. The values represented are mean (standard error) of the peptide spectral match numbers of the five samples analyzed per group (normal or NASH). *p* value represents difference in mean values between the two conditions

## Discussion

The aim of our pilot study was to identify putative differences in the HDL proteome between morbidly obese individuals with different liver pathologies, and whether these differences suggest a potential change in HDL function. Such a change in function of HDL particles suggested through proteomic analysis may help in the assessment and treatment of cardiovascular disease risk in NAFLD patients. The development of obesity and dyslipidemia is associated with increased CVD risk and is inversely correlated with HDL particle sizes [4, 5]. While studies have shown specific protein and functional changes in HDL particles in diabetes, obesity, and CVD, no study to date investigated whether characteristic changes in the HDL protein composition in patients with NAFLD and NASH may suggest functional changes relevant to the increased CVD risk.

In our exploratory proteomic analysis of HDL particles isolated from NAFLD patients, we identified 95 putative HDL-associated proteins. Similar proteomic analyses of HDL particles have identified on average 85–100 proteins [18]. The HDL protein cargo is a dynamic feature of HDL particles and reflects their function and physiological status. Thus, the number of protein identifications is similar to what has been reported by other groups and includes a large proportion of proteins routinely identified in HDL particles. Additional proteins identified in our analysis that are not routinely reported as HDL-associated include predominantly immunoglobulin fragments, complement-related proteins, and coagulation factors. We also identified attractin (ATRN) which is involved in inflammatory response, vitamin K dependent protein S (PROS), an anticoagulant, and corticosteroid binding globulin (CBG), a serine protease inhibitor in our HDL analysis. Since the subjects in this study are all morbidly obese, the proteins may also reflect obesity related-proteins in HDL particles. For example, CBG was identified as an inflammatory marker and stress response protein in obese patients with hypertension [19].

We recognize the concern that SEC method of HDL isolation and subsequently the identification of HDL proteome may not completely agree with gold standard methods such as density gradient ultracentrifugation, electrophoretic separation or antibody mediated methods. Our findings indicate that our enrichment procedure likely still retains some co-eluting serum protein complexes that may not be directly associated with HDL particles. However, the number of HDL-associated proteins, and their relative abundance, suggests that the analyzed serum fraction is significantly enriched for HDL particles. Lipoprotein separation by SEC has been used in other studies for the analysis of both lipids [20] and proteins [21] and has been shown to be comparable to separations obtained by ultracentrifugation [22]. The proteins that we observe to be significantly different between normal and NAFLD subjects are known constituents of HDL particles based on previous studies. HDL isolation by SEC may identify unique proteins compared to density gradient ultracentrifugation and certain proteins may not overlap with traditional HDL isolation methods. It will be an interesting avenue in future studies to compare the HDL proteome affected in NASH/NAFLD subjects when the HDL particles are isolated using techniques other than SEC.

Our comparison of protein abundances between the three conditions identified 12 nominally significantly different proteins. Three serpin family proteins (ANT3, CBG and AACT) were significantly decreased in subjects with SS and NASH compared with subjects with normal livers. ANT3 was reported to be differentially expressed in NASH in a recent gene expression analysis of liver samples [23], matching our proteomic findings.

Alpha-2-macroglobulin (A2MG) and APOB were two of the 12 proteins that increased in NASH and SS subjects. A2MG has been used as a marker for fibrogenesis in NAFLD patients (Fibrosure) [24]. Our data confirm increased levels of A2MG in NAFLD patients.

Interestingly, APOB has been predominantly studied in the context of VLDL or LDL particles. Our group and others have shown that APOB can be identified as an HDL-associated protein [6, 8, 10]. Our study shows a positive correlation between APOB abundance in HDL particles and NAFLD severity. mRNA expression levels of *APOB* are significantly increased in NAFLD patients [25].

PON1 levels and activity have been reported to be reduced in NAFLD patients, and PON1 has been suggested as a biomarker for diagnosis of NASH [26]. Interestingly, in our study, PON1 showed no significant difference in abundance in NASH and SS subjects when compared to samples obtained from obese subjects with normal liver pathology.

Our comparison of HDL-associated protein abundances between the three conditions only shows small fold changes. This study was conducted between subjects who have high BMI (mean = 49.5 kg/m^2^) and the confounding variable was the presence or absence of steatosis. We believe that this comparison resulted in more subtle differences in protein abundances between the HDL proteomes, in addition to already significant alterations due to the morbid obesity of the patients. Investigation of qualitative differences between the conditions did not identify any proteins unique to any one condition, suggesting that protein differences in HDL particles between morbidly obese individuals only change quantitatively with the development of additional liver pathologies. It remains to be seen whether any of these proteomic changes can be validated in larger studies to serve as potential biomarkers for NAFLD or NASH.

Apart from RCT, HDL particles also function as anti-inflammatory, anti-oxidant and anti-thrombotic agents since they carry a variety of proteins mediating these functions. Gene ontology analysis in the “biological processes” (BP) and “molecular function” (MF) categories of HDL proteins significantly different between patients with normal liver pathology and NAFLD patients (SS *plus* NASH) indicated an over-representation of GO terms related to coagulation and endopeptidase inhibitor activity. Combining abundance data with GO term over-representation analysis indicates that the anti-thrombotic function of HDL proteins may be altered in NAFLD. ANT3 is the only protein which showed significantly decreased abundance between normal, SS and NASH patients as well as normal versus NAFLD patients. ANT3 is an important anti-coagulant that is activated by heparin [27]. HEP2 is also decreased in NAFLD, indicating that HDL particles from affected patients may mediate prothrombotic functions rather than serving as anti-thrombotic particles, increasing CVD risk.

In this study, we decided to not correct for type-1 errors. First, the study was designed as a pilot analysis with a small sample size. Second, since the true relationship of the identified proteins is not known, a traditional stringent multiple testing correction would remove true biologically relevant proteins as false negatives from the data. We recognize that without this correction certain proteins identified may be construed as false positives in our analysis. However, gene ontology enrichment analysis of the data implicates a specific set of proteins involved in thrombotic function to be over-represented rather than a random set of proteins indicating that the proteins involved in the changes reflect a potential biological phenomenon affected in NAFLD patients. Only more stringent analyses on larger datasets will help resolve whether any of the individual proteins detected in our pilot study would be suitable as a potential biomarker for CVD risk in NASH patients, this clearly exceeded the scope and aims of this initial pilot project.

None the proteins changing with NAFLD and NASH were correlated with the size of HDL particles in our patients (data not shown). Smaller HDL particles are prevalent in subjects diagnosed with morbid obesity and with NAFLD [4]. Our analysis revealed no correlation of protein abundance with HDL particle size, suggesting that the particle size reduction in NAFLD is likely mediated by changes in lipid composition. Also, the additional reduction in HDL particle size in patients with NAFLD and NASH is relatively minor, and morbid obesity alone is correlated with a much larger reduction in HDL particle size. Therefore, the effect of altered protein abundances on particle size may not be detectable in our study.

Our analysis of the serum proteome from the same samples strongly suggests that these observed differences are not merely a reflection of differences existing in the serum. In fact, our analysis shows that none of the proteins identified in the serum are significantly different in abundance between samples from individuals with normal liver function and samples from individuals with NAFLD or NASH. Specifically, proteins that show functional roles related to blood coagulation or anti-thrombotic functions were not found to be significantly different in the serum proteome between normal and NASH subjects (Table 3). This proves that the observed differences are likely related to the HDL particles enriched in the SEC fraction analyzed, and not due to co-eluting serum proteins.

To comprehensively understand and delineate the mechanisms associated with altered HDL proteome and cardioprotective function, further validation of changes in the proteome through orthogonal techniques will be essential to establish the magnitude and statistical relevance of the observed changes. Considering the modest abundance changes we observed in this study, larger cohorts will have to be investigated to confirm or refute the suggestive changes in anti-thrombotic proteins, and their impact on CVD risk in NASH. However, the pilot data presented here, for the first time, provide a putative functional link between HDL particle composition and CVD risk that warrants further study.

## Conclusions

The goal of our proteomic analysis was to characterize differences between proteins associated with HDL particles in morbidly obese patients who have been diagnosed with SS or NASH or have normal liver physiology. We identified several HDL-associated proteins that are significantly changed in NAFLD (SS *plus* NASH). The abundances of these proteins change with disease severity. Quantitative analysis of altered proteins revealed potential changes in HDL function which may reduce anti-thrombotic properties of HDL particles, thereby increasing CVD risk. Currently, there is no reliable analytical method to measure anti-thrombotic properties of isolated HDL particles, thus the proposed functional impact cannot be validated at this time [3]. Further studies will be needed to validate these initial findings, and verify and assess the potential clinical impact of these proteomic changes in patients diagnosed with morbid obesity and NAFLD. Risk towards CVD development in such patients may be exacerbated due to potential altered HDL function.

## Additional file

**Additional file 1.** Proteome Abundance Data

## Abbreviations

NAFLD: non-alcoholic fatty liver disease
NASH: non-alcoholic steatohepatitis
TZD: thiazolidinediones
ALT: alanine transaminase
HOMA: Homeostasis Model Assessment
SEC: size exclusion chromatography
MS: mass spectrometry
SS: simple steatosis
LFQ: label-free quantitation
BP: biological process
MF: molecular function
PSM: peptide spectral match
RCT: reverse cholesterol transport
